# Differential gene expression pattern in early gastric cancer by an integrative systematic approach

**DOI:** 10.3892/ijo.2012.1621

**Published:** 2012-09-06

**Authors:** SEUNGYOON NAM, JIEUN LEE, SUNG-HO GOH, SEUNG-HYUN HONG, NAALEUM SONG, SANG-GEUN JANG, IL JU CHOI, YEON-SU LEE

**Affiliations:** 1 Cancer Genomics Branch; 2 New Experimental Therapeutics Branch; 3 Colorectal Cancer Branch and; 4 Gastric Cancer Branch, Research Institute, National Cancer Center, Goyang, Gyeonggi-do 410-769, Republic of Korea

**Keywords:** estrogen receptor α, gastric cancer, metastasis, microarray, metalloproteinases

## Abstract

To elucidate the molecular basis of early gastric cancer (EGC), the genome-wide expression pattern of cancer and normal tissues from 27 patients were analyzed by a microarray-based method. Using an integrative systematic bioinformatics approach, we classified the differentially expressed genes in EGC. Interestingly, the more highly expressed genes in EGC exhibited the most significant correlation with cell migration and metastasis. This implies that, even at the early stage of gastric cancer, the molecular properties usually observed in late-stage cancer are already present. Furthermore, we have found a novel association between the expression pattern and molecular pathways of EGC and estrogen receptor α (ERα)-negative breast cancer through cross-experimental analysis. These results provide new insights into the biological properties of EGC, as well as yielding useful basic data for the study of molecular mechanisms of EGC carcinogenesis.

## Introduction

Gastric cancer (GC) is the fourth most common cancer and the second leading cause of cancer-related deaths worldwide. Its prevalence is particularly high in East Asia, including countries such as China, Japan and Korea (1). The prognosis of GC depends on the stage of diagnosis, as an early gastric cancer (EGC) or advanced gastric cancer (AGC) (2). Despite the surgical advances that have improved long-term survival of GC patients (3,4), molecular understanding of, as well as novel molecular biomarkers for, the condition is still urgently required for EGC, as EGC may progress towards AGC (2).

To address this, several microarray analyses in GC have been performed and have identified gene expression patterns that may be useful in the prognosis and diagnosis of the cancer (5,6); however, these approaches did not consider the different stages or subtypes of GC. Recent studies that did consider stage differences (2,7,8) did not reveal the multiple phenotypes underlining EGC, because their primary aim was to study a handful of gene sets, which differentiate the stage differences. Accordingly, we further explored the various hidden phenotypes, functions and pathways in EGC by using an integrative systematic bioinformatics approach.

Here, we focus on molecular understanding of EGC-specific expression patterns gained by employing a systematic approach, including function and pathway, as well as cross-experiment analyses of 27 pairs of EGC tissues and their normal counterparts. Interestingly, the function and pathway analyses show that the upregulated genes in EGC tissues correlate with cell migration and metastasis, events typical of late-stage cancer. In addition, we propose a novel association between EGC and estrogen receptor α (ERα)-negative breast cancer that was indicated by cross-experiment analysis, and which enables us to identify various associated phenotypes.

## Materials and methods

### Patients and samples

Tissue samples were prospectively collected from patients who underwent gastric surgery or gastroscopy at the National Cancer Center (NCC) Hospital between 2008 and 2009. All tissues were obtained according to the protocols approved by the Institutional Review Board, NCC for the human subject guideline of NCC (NCCNCS-08-127) that is in accordance with the principles of the Declaration of Helsinki. The samples were obtained by endoscopic biopsies from gastric cancer patients who gave informed consent to the protocol. The samples were stored at –80°C. The clinical and pathological features of the patients are listed in Table I.

### RNA extraction

Total RNA was extracted from gastric cancer and adjacent normal tissues from EGC patients using TRIzol reagent (Invitrogen, Carlsbad, CA, USA), followed by purification of the RNA using Qiagen RNeasy mini kit columns (Valencia, CA, USA) according to the manufacturer’s instructions. RNA quality was evaluated using the Agilent 2100 Bioanalyzer (Agilent Technologies, Palo Alto, CA, USA) and concentration measured by Nanodrop 1000 (Thermo Scientific, Wilmington, DE, USA). Only RNAs showing distinct 18S/28S ribosomal peak ratios of 1.5–2.0 in the Bioanalyzer (Agilent Technologies) and 260/280 ratios of 1.8–2.1 in the Nanodrop (Thermo Scientific) analyses were accepted for further analysis.

### Microarray analysis and data processing

Genome-wide gene expression was analyzed in the 27-paired EGC tissue samples using Affymetrix GeneChip Human Exon1.0 ST Array (Santa Clara, CA, USA). Target preparation and microarray processing procedures were carried out as described in the manufacturer’s instructions, and raw data were deposited in the NCBI Gene Expression Omnibus (GSE30727). The data were preprocessed by a default robust multi-array average (RMA) method implemented in the Bioconductor (www.bioconductor.org) ‘oligo’ package. The differentially expressed genes between EGC tissues and adjacent non-cancerous gastric tissues (i.e., the up- and downregulated genes in EGC) were filtered by a fold-change cut-off of 1.5 and a P-value cut-off of 0.05.

### Functional/pathway enrichment analysis and cross-experimental analysis

We downloaded a Gene Ontology (GO) annotation file (gene_association.goa_human) and an ontology file (gene_ontology_ext.obo) from www.geneontology.org, as recommended by the BiNGO tutorial (9). In the BiNGO analysis, all options, except for filtering the IEA code, were set at default values. The false discovery rate (FDR) cut-off was 0.05. DAVID v6.7 software (http://david.abcc.ncifcrf.gov/) was used to summarize the over-representation of the KEGG pathways (10). The gene expression signatures of up- or downregulated genes in EGC were analyzed using the L2L microarray analysis tool (http://depts.washington.edu/l2l/) (11).

### Reverse transcription PCR

Two micrograms of total RNA were reverse transcribed with Superscript III reverse transcriptase (Invitrogen). Reverse transcription PCR (RT-PCR) was performed using 5 ng cDNA for 1 cycle at 94°C for 2 min, followed by 32–35 cycles of 94°C for 20 sec, 60°C for 40 sec and 72°C for 30 sec, using gene-specific primers (Table II). Gene expression levels were analyzed by gel electrophoresis.

### Hierarchical clustering

Independent additional cancer datasets were obtained from NCBI GEO (www.ncbi.nlm.nih.gov/geo) and EBI ArrayExpress (www.ebi.ac.uk/arrayexpress): GSE19536 for ERα-negative breast cancers (12), and the E-MTAB-62 dataset for Ewing’s sarcoma, bladder cancer, small cell lung cancer and LNCaP prostate cancer cell lines (13). The up- and downregulated genes in the EGC tissues were compared with these 5 cancer types. We transformed the expression of all our EGC tissue samples, GSE19536 and E-MTAB-62, into standard scores (z-scores), and then performed hierarchical clustering for the 6 cancers.

## Results

### Genome-wide expression analysis

We selected differentially expressed genes (i.e., up- and downregulated genes) from the 27 pairs of EGC tissue and their adjacent normal tissue. The P-value cut-off of 0.05 in t-tests, and the fold-change cut-off of 1.5 or 1/1.5 for up- and downregulated genes, respectively, was used for selection. We identified 556 upregulated genes and 417 downregulated genes. The differentially expressed genes were then fed into function, pathway and cross-experiment analyses to acquire a deeper understanding of the molecular basis of EGC.

### Functional enrichment analysis

The BiNGO plug-in on the Cytoscape platform (http://www.cytoscape.org/) was used to explore the molecular function and biological processes in GO.

**Figure 1.**
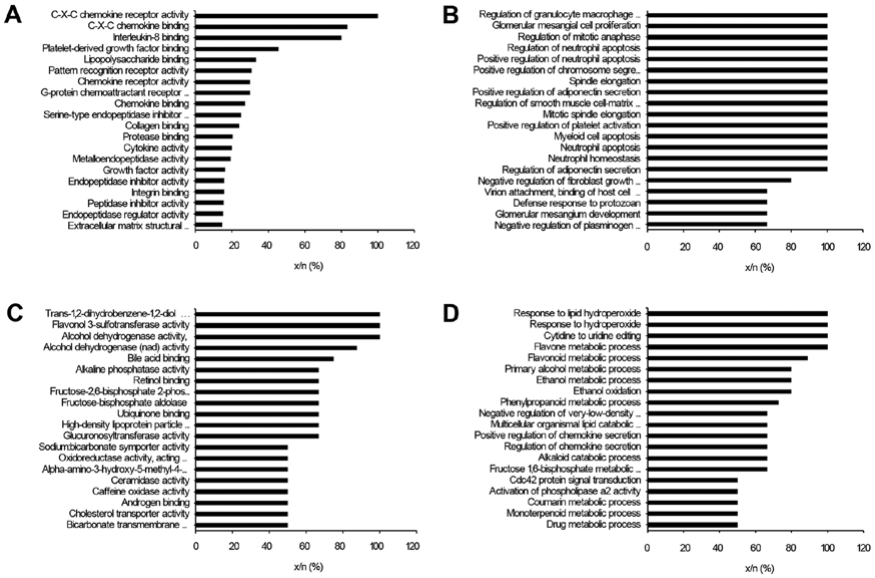
The GO analysis by BiNGO. (A) The upregulated molecular functions in EGC tissues. (B) The upregulated biological process in EGC tissues. (C) The downregulated molecular functions in EGC tissues. (D) The downregulated biological process in EGC tissues. The Information is presented as a percentage of x/n (x, the number of genes in a cluster annotated for a certain GO term; n, the number of genes in the reference set annotated for a certain GO term).

The functions of the upregulated genes of EGC tissues were significantly associated with C-X-C and other chemokine-related signaling, interleukin-8 binding, growth factor binding, collagen binding, and the extracellular matrix (ECM) (Fig. 1A). Moreover, the biological processes involved in the upregulated genes in the EGC tissues were strongly related to cell proliferation, mitosis, apoptosis and cell-matrix adhesion (Fig. 1B). Additionally, wound healing terms, cell migration terms and cell motility terms were also listed in the upregulated genes with statistical significance (Table III). Since cell migration, cell motility and wound healing are typically observed in late-stage, metastatic cancer, this may indicate that EGC tissues could possess intrinsic aggressiveness, despite their early detection. Conversely, the downregulated genes were strongly linked to oxidoreductase activity (e.g., oxidoreductase activity acting on the CH or CH2 groups, quinones) in GO molecular function (Fig. 1C). Furthermore, the downregulated genes were enriched in various terms related to metabolic processes (e.g., flavone and flavonoid metabolic pathways) in GO biological processes (Fig. 1D). The GO terms of the downregulated genes clearly indicate dysregulation of metabolism in EGC, which is one of the emerging cancer hallmarks (14).

### Pathway enrichment analysis

The DAVID tool (http://david.abcc.ncifcrf.gov/) was used to inspect the KEGG biological pathways associated with the differently expressed genes in EGC.

The upregulated genes in EGC tissues were intrinsically associated with cytokine-cytokine receptor interactions, ECM-receptor interactions, the cell cycle, hematopoietic cell lineage and Toll-like receptor signaling pathways (Table IV). In addition, focal adhesion and cell adhesion molecule pathways were highlighted. Thus, similar to the functional enrichment analysis, upregulated pathways in these tumor tissues suggest a strong potential for cell motility and metastasis, despite early detection. In contrast, the downregulated genes in the EGC tissues were strongly associated with xenobiotics-, drug-, retinol-, starch- and sucrose-related metabolism, steroid hormone biosynthesis, as well as pentose and glucuronate interconversion pathways (Table IV).

### The expression of MMPs in EGC tissue

Our functional and pathway analyses demonstrated that the significantly upregulated genes in EGC tissues are associated with cell migration and metastasis, events typical of late-stage cancer. To verify our findings, we further analyzed the expression pattern of matrix metalloproteinases (MMPs), which are well known cell migration-related genes. MMPs are also known to play critical roles in the regulation of cell invasion by ECM proteolysis, as well as by processing cytokine precursors in the chemokine network.

**Figure 2.**
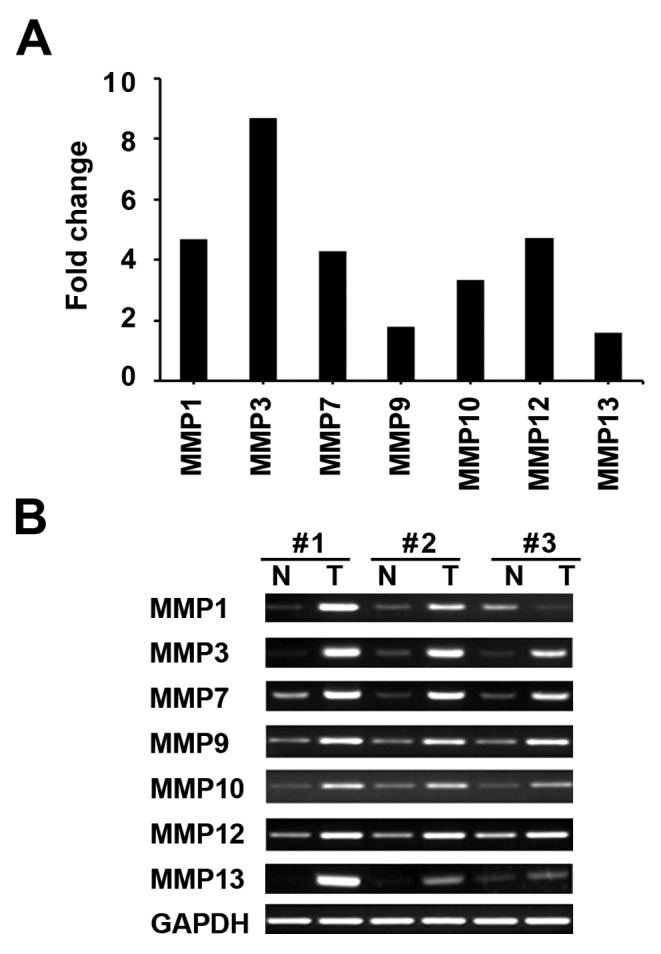
The expression of MMPs in EGC tissues. (A) Expression levels of MMPs genes in microarray data. The vertical axis represents fold-change of the cancer tissues over normal tissues. (B) mRNA expression of MMPs using RT-PCR. Three pairs of non-cancerous and tumor tissues from the microarray analysis were used. (N, adjacent non-cancerous gastric tissue; T, EGC tissue).

We analyzed the expression pattern of 7 MMPs (MMP1, −3, −7, −9, −10, −12 and −13) within the upregulated gene data in EGC tissues. As expected, and consistent with the microarray data where MMPs were upregulated 1.56- to 8.68-fold (Fig. 2A), RT-PCR indicated that MMP mRNA expression was highly upregulated in the patients’ EGC tissues (Fig. 2B).

### Cross-experimental analysis

In order to investigate similar molecular signatures between EGC and other cancer types, we compared our data of differentially expressed genes with a public gene expression signature warehouse, L2L. This revealed that the upregulated genes in EGC most significantly correlated with the gene expression signature of ERα-negative breast cancer (Table V). As summarized in Table V, the upregulated genes in EGC were also similar to the gene expression signature related to an undifferentiated cancer status (cancer_undifferentiated_meta_up: 69 genes commonly upregulated in undifferentiated cancer relative to well-differentiated cancer, from a meta-analysis of the OncoMine gene expression database), stemness (stemcell_embryonic_up: enriched in mouse embryonic stem cells, compared to differentiated brain and bone marrow cells) and survival (dox_resist_gastric_up: upregulated in gastric cancer cell lines resistant to doxorubicin, compared to parent chemosensitive lines). Together, the EGC tissues reflect various facets of cancer-related phenotypes, viz., strong survival, stem-like and morphology.

The same L2L analysis was applied to the downregulated genes in EGC (Table V). Interestingly, epigenetic-related cancer gene expression signature terms (5azac_hepg2_up and 5azac-tsa_hepg2_up in Table V) were highly ranked. This suggests that global alterations in DNA methylation and histone modification occur in EGCs, as it does in other cancers.

### Hierarchical clustering of the EGC tissues and other cancers

**Figure 3.**
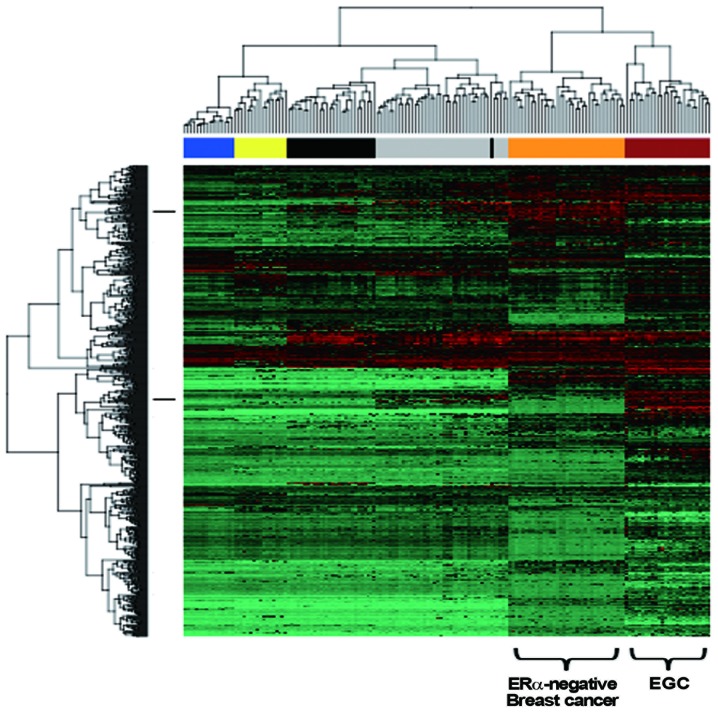
Hierarchical clustering. Genes up- or downregulated in EGC as well as 5 independent cancer types were used in the hierarchical clustering analysis. Each cancer type is presented with the following column side-bars: EGC (brown), ERα-negative breast cancers (orange), bladder cancer (grey), Ewing sarcoma (black), small cell lung cancers (yellow) and LNCaP prostate cancer cell lines (blue). Seven MMP genes are presented with row side-bars.

To validate the result of the L2L analysis showing a relationship between EGC and ERα-negative breast cancer, we performed a hierarchical clustering analysis. The expression datasets of the differently expressed genes in EGC (556 upregulated gene symbols and 417 downregulated gene symbols), ERα-negative breast cancer and 4 additional cancers (small cell lung cancer, LNCaP prostate cancer cell lines, bladder cancer and Ewing sarcoma) were used in an unsupervised hierarchical clustering analysis. As in the L2L analysis, the results indicated that EGC correlated most closely with the ERα-negative cancer than with the other 4 cancers (Fig. 3).

**Figure 4.**
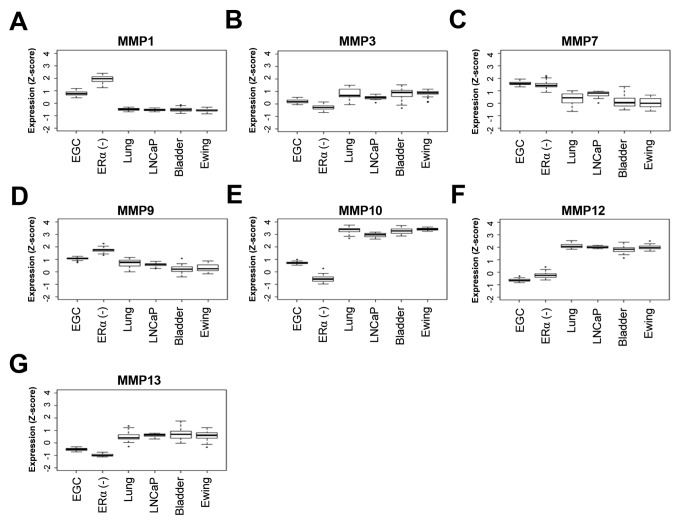
The expressions (Z-scores) of 7 MMP genes. Z-score zero corresponds to the mean of all expressions in each cancer type. (EGC, the EGC tissues; ERα (-), ERα-negative breast cancer; Lung, lung small cell cancer; LNCaP, LNCaP prostate cancer cell lines; Bladder, bladder cancer; and Ewing, Ewing sarcoma).

When we inspected the expression levels (z-scores) of the 7 MMP genes (Fig. 4), the results indicated that the ERα-negative cancer, above all other observed cancers, showed the most similar expression patterns for the 7 MMPs. Overall, the hierarchical clustering was consistent with the cross-experimental analysis and strongly supported the molecular similarity between EGC and ERα-negative breast cancer in terms of carcinogenesis.

## Discussion

We analyzed the microarray data generated from pairs of tumor tissue and their adjacent non-cancerous tissue, obtained from 27 EGC patients. The gene expression data were subjected to functional and pathway analyses, as well as gene expression signature comparison (cross-experiment analysis). This led to 2 novel findings: i) the functional and pathway analyses suggested that metastasis-related biological processes may already be highly expressed even in the early stage of gastric cancer, and ii) the gene expression pattern of EGC is closely aligned to that of ERα-negative breast cancer.

We also compared the differentially expressed genes in our EGC tissues with other 3 previously published gene expression studies (2,7,8). We found that the upregulated genes in our study significantly overlapped with the upregulated genes in the EGC groups of the 3 earlier studies (2,7,8), under a randomization model (Table VI). Recently, Vecchi *et al* suggested a carcinogenesis model (2) in which the transition from normal mucosa to EGC is accompanied by cell cycle upregulation; our pathway analysis results (hsa04110, cell cycle in Table IV) is consistent with this model. Interestingly, AGC functions (cell migration-and ECM-related functions), suggested by the Vecchi model were also revealed in our EGC data, again indicating that EGC actually harbors gene expression events that are usually observed in the later stages of cancer, such as AGC.

Based on our functional and pathway analyses, the upregulated genes in the EGC tissues were highly enriched for genes involved in cell proliferation, chemokine/growth factor signaling and cell migration. The computational implication is, in fact, closely related to MMP activity, as MMP substrates include growth factor/chemokine precursors and E-cadherin (15,16). We validated the upregulation of the 7 MMPs in the EGC tissues by RT-PCR. This result suggests that the activation of multiple MMPs may be involved in the early stage of cancer. The suggestion is noteworthy, when considering that the roles of multiple MMPs were mainly reported in late-stage gastric cancer (2,17). It is also interesting to note that 6 (MMP1, −3, −7, −10, −12 and −13) of the 7 MMPs are clustered at 11q22, implying that epigenetic events could be involved in the upregulation of the clustered MMPs (18).

**Figure 5.**
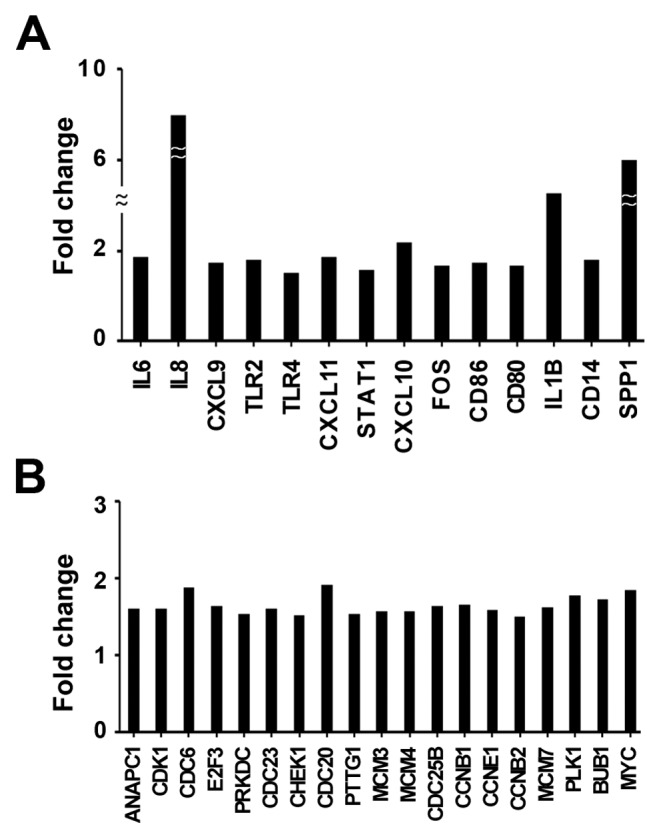
The upregulated genes belonging to the KEGG TLR signaling pathway (A) and cell cycle pathway (B) in our EGC tissues.

Additionally, we found that the gene expression pattern in EGC tissues resembles the pattern of the ERα-negative breast cancer transcriptome. Since ERα-negative breast cancer clusters with EGC (Fig. 3), the similarity suggests that these two cancers may share common molecular features. Recent breast cancer studies (19,20) reported that high expression of cyclooygenase-2 (Cox-2), encoded by PTGS2, is associated with poor survival in ERα-negative breast cancer patients, when compared to ERα-positive breast cancers. Interestingly, Cox-2 is highly involved in the inflammation-associated carcinogenesis of the gastrointestinal tract. In particular,

*H. pylori*-infected gastric epithelial cells can experience malignant transformation via Toll-like receptor (TLR) signaling that induces Cox-2, followed by activation of cell proliferation (21). In fact, our pathway analysis in EGC showed upregulation of the KEGG TLR signaling pathway and cell cycle pathway (Table IV, Fig. 5). Our EGC also showed a markedly increased expression of PTGS2 (5.74-fold-change). Thus, the similarity between EGC and ERα-negative breast cancer may come from identical subsets of immune response-related signaling between the microenvironments of the tumors.

In conclusion, we have analyzed the differentially expressed genes in EGC patients using an integrative systematic approach. We found that genes highly expressed in EGC are involved in cell migration- and metastasis-related functions typically observed in late-stage cancer. Also, EGC may be intrinsically similar to ERα-negative breast cancer, by sharing immune-related signaling events, which is further dissected in both cancer types. The functional roles of the downregulated genes in EGC carcinogenesis remain to be elucidated in future.

## Figures and Tables

**Table I.** Clinical features for 27 patients with gastric cancer.

Characteristics	No. of patients^a^
Total	27
Male	20
Female	7
Age at diagnosis (years)	
Range	41–78
Mean ± SD	60.3±11.2
TNM stage^b^	
T classification	
T1	17
T2	10
T3	0
N classification	
N0	13
N1	10
N2	2
N3	2
M classification	
M0	27
M1	0
Lauren classification	
Intestinal	12
Diffuse	7
Mixed	6
NA^c^	2

a The 27 patient samples were used in microarray analysis.

b UICC/AJCC 6th edition.

c Not available of Lauren classification.

**Table II.** The primer sequences used in RT-PCR.

Primer ID	Sequence (5′→3′)
MMP1-F	CTGGAATTGGCCACAAAGTT
MMP1-R	CCTTCTTTGGACTCACACCA
MMP3-F	CCCTGGGTCTCTTTCACTCA
MMP3-R	TCAAAGGACAAAGCAGGATC
MMP7-F	CGGATGGTAGCAGTCTAGGG
MMP7-R	TGAATGGATGTTCTGCCTGA
MMP9-F	GGGAAGATGCTGCTGTTCA
MMP9-R	TCAACTCACTCCGGGAACTC
MMP10-F	GGCTCTTTCACTCAGCCAAC
MMP10-R	TCCCGAAGGAACAGATTTTG
MMP12-F	CCTTCAGCCAGAAGAACCTG
MMP12-R	ACACATTTCGCCTCTCTGCT
MMP13-F	TTGAGCTGGACTCATTGTCG
MMP13-R	GGAGCCTCTCAGTCATGGAG
GAPDH-F	TGCACCACCAACTGCTTA
GAPDH-R	GGATGCAGGGATGATGTTC

**Table III.** The GO biological process terms associated with genes upregulated in gastric cancer tissues, relating to wound healing, cell migration and cell motility.

GO-ID	P-value	Corrected P-value^a^	x^b^	n^c^	x/n (%)	Description
GO:0014910	1.54E-03	1.86E-02	4	14	29	Regulation of smooth muscle cell migration
GO:0061041	5.41E-07	3.15E-05	11	44	25	Regulation of wound healing
GO:0010595	4.41E-03	4.25E-02	5	29	17	Positive regulation of endothelial cell migration
GO:0030335	3.69E-07	2.38E-05	18	116	16	Positive regulation of cell migration
GO:2000147	3.69E-07	2.38E-05	18	116	16	Positive regulation of cell motility
GO:0030334	9.12E-07	4.44E-05	23	190	12	Regulation of cell migration
GO:2000145	1.44E-06	6.12E-05	23	195	12	Regulation of cell motility
GO:0048870	9.75E-10	1.81E-07	38	330	12	Cell motility
GO:0042060	1.04E-10	3.09E-08	50	485	10	Wound healing

a Multiple comparison corrected P-value.

b The number of the input genes annotated to a certain GO term.

c The number of genes in the reference set annotated to a certain GO term.

**Table IV.** Pathway enrichment analysis for up- and downregulated genes in gastric cancer tissues.

Input genes	Pathways	Count^a^	P-value
Upregulated pathways	hsa04060, cytokine-cytokine receptor interaction	38	3.61.E-11
	hsa04512, ECM-receptor interaction	17	3.16.E-07
	hsa04110, cell cycle	19	4.21.E-06
	hsa04640, hematopoietic cell lineage	13	2.40.E-04
	hsa04620, Toll-like receptor signaling pathway	14	3.02.E-04
	hsa04062, chemokine signaling pathway	20	3.18.E-04
	hsa04610, complement and coagulation cascades	11	5.85.E-04
	hsa04510, focal adhesion	19	2.00.E-03
	hsa04115, p53 signaling pathway	10	2.11.E-03
	hsa04514, cell adhesion molecules (CAMs)	14	3.72.E-03
	hsa05222, small cell lung cancer	10	8.79.E-03
	hsa04670, leukocyte transendothelial migration	12	1.11.E-02
	hsa05020, prion diseases	6	1.51.E-02
	hsa04621, NOD-like receptor signaling pathway	8	1.53.E-02
	hsa05200, pathways in cancer	23	2.09.E-02
	hsa05332, graft vs. host disease	6	2.34.E-02
	hsa05322, systemic lupus erythematosus	10	2.40.E-02
	hsa05219, bladder cancer	6	3.13.E-02
	hsa04114, oocyte meiosis	10	4.32.E-02
	hsa04650, natural killer cell mediated cytotoxicity	11	5.53.E-02
	hsa04142, lysosome	10	5.97.E-02
	hsa04630, Jak-STAT signaling pathway	12	6.43.E-02
	hsa04914, progesterone-mediated oocyte maturation	8	7.17.E-02
Downregulated pathways	hsa00980, metabolism of xenobiotics by cytochrome P450	22	3.33.E-18
	hsa00982, drug metabolism	22	7.29.E-18
	hsa00830, retinol metabolism	19	2.76.E-15
	hsa00140, steroid hormone biosynthesis	13	3.63.E-09
	hsa00500, starch and sucrose metabolism	11	2.00.E-07
	hsa00040, pentose and glucuronate interconversions	8	3.57.E-07
	hsa00590, arachidonic acid metabolism	12	3.95.E-07
	hsa00983, drug metabolism	10	2.69.E-06
	hsa00053, ascorbate and aldarate metabolism	7	5.02.E-06
	hsa00591, linoleic acid metabolism	8	1.04.E-05
	hsa00860, porphyrin and chlorophyll metabolism	8	3.32.E-05
	hsa00010, glycolysis/gluconeogenesis	10	4.64.E-05
	hsa00150, androgen and estrogen metabolism	8	7.27.E-05
	hsa03320, PPAR signaling pathway	7	1.41.E-02
	hsa00051, fructose and mannose metabolism	5	1.59.E-02
	hsa00330, arginine and proline metabolism	6	1.78.E-02
	hsa00071, fatty acid metabolism	5	2.74.E-02
	hsa00030, pentose phosphate pathway	4	3.42.E-02
	hsa00350, tyrosine metabolism	5	3.72.E-02
	hsa00920, sulfur metabolism	3	4.53.E-02
	hsa00340, histidine metabolism	4	5.00.E-02
	hsa00480, glutathione metabolism	5	5.54.E-02

a Count represents the number of input genes assigned to the KEGG pathway.

**Table V.** The selected L2L results of genes up- or downregulated in early gastric cancer.

Input genes	L2L term (PubMed ID)	Description	x^a^/n^b^	Binomial P-value
Upregulated genes in the gastric cancer tissues	brca_er_neg (11823860)	Genes whose expression is consistently negatively correlated with the gastric cancer tissues estrogen receptor status in breast cancer - higher expression is associated with ER-negative tumors	145/996	7.50E-95
	cancer_undifferentiated_meta_up (15184677)	Sixty-nine genes commonly upregulated in undifferentiated cancer relative to well-differentiated cancer, from a meta-analysis of the OncoMine gene expression database	25/69	2.21E-28
	dox_resist_gastric_up (14734480)	Upregulated in gastric cancer cell lines resistant to doxorubicin, compared to parent chemosensitive lines	17/48	1.44E-19
	stemcell_embryonic_up (12228720)	Enriched in mouse embryonic stem cells, compared to differentiated brain and bone marrow cells	74/1350	1.32E-21
Downregulated genes in the gastric cancer tissues	5azac_hepg2_up (16854234)	Upregulated in human hepatoma cells (HepG2) following 48 of treatment with 2.5 *μ*M 5-aza-2-deoxycytidine (5azaC)	60/1311	6.61E-20
	5azac-tsa_hepg2_up (16854234)	Upregulated in human hepatoma cells (HepG2) following 24 h of treatment with 2.5 *μ*M 5-aza-2-deoxycytidine (5azaC) and 24 h of treatment with both 5azaC and 500 nM trichostatin A (TSA)	66/1619	3.69E-19

a The number of the input genes annotated to a L2L term.

b The number of genes in the reference set annotated to a L2L term.

**Table VI.** Comparisons with other GC sources in terms of upregulated EGC-related genes.

Refs.	EGC classification	n^a^	x^b^	Significance (P-value)^c^
(2)	-	488	83	<2.2E-16
(7)	Well-differentiated (WD) and moderately differentiated (MD)	170	62	<2.2E-16
(8)	AJCC staging I and II (TNM staging)	118	15	1.601E-06

a The number of upregulated genes in the cancer according to the references.

b The number of common upregulated genes (intersection) between the references and ours.

c The significance of the intersections between our EGC upregulated genes and the studies were calculated. Fisher’s exact test, based on the randomization model, was used to obtain the P-values of the intersections from a hyper-geometric distribution. The smaller the P-value, the more significant the agreement between the previous study and our EGC study. The total number of gene symbols used in the Fisher’s exact test is 19,211 (HUGO Gene Nomenclature Committee).
